# A Biodiverse Rich Environment Does Not Contribute to a Better Diet: A Case Study from DR Congo

**DOI:** 10.1371/journal.pone.0030533

**Published:** 2012-01-24

**Authors:** Céline Termote, Marcel Bwama Meyi, Benoît Dhed'a Djailo, Lieven Huybregts, Carl Lachat, Patrick Kolsteren, Patrick Van Damme

**Affiliations:** 1 Laboratory of Tropical and Subtropical Agronomy and Ethnobotany, Faculty of Bioscience Engineering, Ghent University, Gent, Belgium; 2 Laboratory of Development Economics and Environment, Institut Facultaire des Sciences Agronomiques - Yangambi, Kisangani, Democratic Republic of Congo; 3 Laboratory of Genetics, Plant Improvement and Biotechnology, Faculty of Science, University of Kisangani, Kisangani, Democratic Republic of Congo; 4 Department of Food Safety and Food Quality, Faculty of Bioscience Engineering, Ghent University, Gent, Belgium; 5 Nutrition and Child Health Unit, Institute for Tropical Medicine, Antwerpen, Belgium; New York State Museum, United States of America

## Abstract

The potential of biodiversity to increase and sustain nutrition security is increasingly recognized by the international research community. To date however, dietary assessment studies that have assessed how biodiversity actually contributes to human diets are virtually absent. This study measured the contribution of wild edible plants (WEP) to the dietary quality in the high biodiverse context of DR Congo. The habitual dietary intake was estimated from 2 multiple-pass 24 h dietary recalls for 363 urban and 129 rural women. All WEP were collected during previous ethnobotanical investigations and identified and deposited in the National Botanical Garden of Belgium (BR). Results showed that in a high biodiverse region with precarious food security, WEP are insufficiently consumed to increase nutrition security or dietary adequacy. The highest contribution came from *Dacryodes edulis* in the village sample contributing 4.8% of total energy intake. Considering the nutrient composition of the many WEP available in the region and known by the indigenous populations, the potential to increase nutrition security is vast. Additional research regarding the dietary contribution of agricultural biodiversity and the nutrient composition of WEP would allow to integrate them into appropriate dietary guidelines for the region and pave the way to domesticate the most interesting WEP.

## Introduction

The inextricable link between biodiversity and nutrition security gains more and more interest from researchers all over the world [1]–[4]. Plant genetic diversity as such, embedding genes for desirable traits, has a critical role to increase and provide sustainable production levels and nutritional diversity [4].

In many areas, Wild Edible Plants (WEP) play a major role in supplementing staples with micronutrients [3], [5] or constitute a ‘safety net’ during periods of food shortage [6], [7]. Various indigenous food systems described in Kuhnlein et al. [8] provide a wide array of nutrients required for good health and well-being. In many of such communities, traditional food products are increasingly being replaced by imported or newly introduced foods, in particular rich in refined and processed foods, fats and sugars [9]. Traditional diets are globally disturbed whereby deficiencies in nutrients [9], [10] and diet-related non-communicable diseases such as obesity, type 2 diabetes and cardio-vascular diseases may emerge [8]. On the other hand, undernutrition and micronutrient deficiencies are a persisting public health challenge in sub-Saharan Africa [9], undermining health, psychological well-being, work capacity and economic development [11]–[13]. The indigenous agricultural potential of sub-Saharan Africa has largely remained unexplored to address the current dietary challenges [14].

To date however, dietary assessment studies that have addressed how WEP and agricultural biodiversity actually contribute to human diets are virtually absent [15]. In particular, few studies are available that combine exact botanical identification of species (especially wild species) with proper quantitative dietary assessment methods. It consequently remains unclear how biodiversity contributes to dietary quality.

As a best case scenario, we carried out our investigations in a high biodiverse environment, in the Democratic Republic of Congo (DRC), estimated to be the 5th most biodiverse country on earth [16]. Despite its enormous agricultural potential, food security remains very precarious [17]. Information on the current nutrient intake in DRC is scant and no previous dietary intake study has focused on the contribution of WEP from a nutritional perspective. However, interesting species, with potential to increase nutrition security, have been reported in the region such as the seeds of the African breadfruit tree (*Treculia africana* Decne. ex Trécul) [18]–[20] or the leafy vegetable ‘fumbwa’ (*Gnetum africanum* Welw.) [21], [22].

The present paper documents the contribution of the WEP to local diets in the city of Kisangani and Yaoseko, a rural village inhabited by the Turumbu. It describes the overall diet in the localities and evaluates the dietary intake of women in the study area in relation to the recommended dietary allowances (RDA) [23], [24]. The present study is part of a broader project that maps all WEP used in the region and studies their nutritional, socio-economic and cultural importance in order to set priorities for participatory domestication and market chain development.

## Materials and Methods

As required by the University of Kisangani, research in the region needs to be approved by the Academic Secretary and his council of the University of Kisangani. The research protocol and the questionnaires have been reviewed prior to data collection, approval number SGAC/145/2009.

Before starting field work, written consent was obtained from the local leaders (Kisangani Mayor and village head of Yaoseko) as stipulated in the approved research protocol. Because participants were mainly illiterate, oral informed consent was obtained. Oral informed consent was confirmed by the supervisors or by a second interviewer acting as witness.

### Sample

The study area comprised the 6 municipalities (Makiso, Tshopo, Lubunga, Kisangani, Mangobo and Kabondo) of Kisangani (capital of the Oriental Province, DRC, 0°31′NB, 25°11′, 428 m) and the rural Turumbu village Yaoseko (34 km West of Kisangani, 0°35′03″N, 24°56′14″E, 400 m). Yaoseko was chosen because it was the village with highest WEP knowledge during previous ethnobotanical inventories in Tshopo district (77 WEP species [25], [26]). The hot and humid climate in the region is classified as Af in Köppen's typology [27]. Kisangani is situated in the Guineo-Congolian regional center of endemism with mixed moist semi-evergreen forests [28]. Data were collected from a cross-sectional survey conducted between July and September 2009 including 241 adult women in Kisangani (all ethnical groups mixed, further indicated as ‘overall city sample’), 129 Turumbu women from the village Yaoseko (further indicated as ‘Turumbu village sample’) and 122 Turumbu women living in the city of Kisangani (further indicated as ‘Turumbu city sample’). Studying actual WEP use and not knowledge, we opted to work with women because of two reasons. Firstly, women take care of cooking and are the decision makers for nutrition in the households in DRC [29]. Recalling food intake from men might induce additional bias and underreporting. Secondly, women are central to address intergenerational effects of malnutrition as their nutrition status not only determines their own health, but also that of future generations through pregnancy and lactation [30]. The research was conducted during the period of highest WEP availability [25], [26]. No reliable inhabitants' lists or population estimates were available for Kisangani so convenience sampling was used. The Kisangani sample was two-stage stratified. For each of the six municipalities of the city a random sample of 4 boroughs was drawn based on the list of boroughs available at the municipality's administration. Starting from a central point (hospital, church, etc.) in each sampled borough, a first household was visited. When an adult woman was at home, research goals and methods were clearly explained and oral consent asked. In case of absence or rejection of participation, the household next door was visited. After each interview, three doors were skipped and the next household visited until reaching the quotum of 10 interviews per borough. This way, 40 women per municipality were selected (41 in Lubunga). For the Turumbu village sample, households were visited from one end of the village to the other end. Per household, we interviewed one adult woman that was home and agreed to participate. As a result 129 on a total of 184 households (70%) were interviewed. For the Turumbu city sample, we relied on the help of the Turumbu *mutuelle* (informal social security system organized by and for the Turumbu living in the city). In absence of addresses, an administrative member of the *mutuelle* assembled Turumbu women at a central point from where the researchers started the interviews until reaching the predefined number. Four interviewers, local women with a university degree, were trained during one week and supervised by 2 researchers. Participants could choose the interview language (Lingala, Kiswahili or French). Uniform translations of the questionnaires in Lingala and Kiswahili were agreed upon between the 4 interviewers during the training sessions. All questionnaires and tools were pre-tested and adapted where necessary.

### Food intake data

Food intake was assessed by two multiple pass 24-h recalls on non-consecutive days [31]. Portion sizes were estimated using, 1) a booklet with photographs of different calibrated portion sizes; 2) an extensive price-weight-conversion list covering all foods or ingredients reported during the 24 h recalls; and 3) direct measurements of estimated leftovers with a digital scale with a precision of 1 g (Soehnle, Nassau, Germany) [32].

Photographs are an appropriate tool to estimate portion sizes at population level in Africa [33], [34]. Portion sizes were calculated as average small, medium and large portion sizes served in local students' and market/road restaurants. The photograph booklet was prepared from the pictures of these average portion sizes as served in locally-used plates.

A price-weight conversion list was composed by visiting the Kisangani central market and 4 municipal markets during the survey period (July–September 2009). Edible portions were weighed using digital dietary scales with a precision of 1 g (Soehnle, Nassau, Germany) and prices recorded for all foods in the 24 h recalls. At least 10 items per food product of the same price category over different vendors per market and in different markets were measured. If necessary, food items were bought to identify edible portion and waste percentages. Consequently, average price-weight conversion factors for use in Kisangani were calculated. Prices in the village did not differ much from those in the city and only for leafy vegetables we needed to calculate adapted conversion factors for use in the village.

Edible portions of fruits and snacks, which come in discrete units, were estimated with the price-weight conversion list. For mixed dishes, we recorded all the ingredients of the total recipe in monetary value and converted them into edible weights by means of the price-weight conversion list. With the aid of the photo-book the total amount of mixed dish cooked (number of plates and respective portion sizes) as well as the amount individually consumed by the interviewee were estimated. In this way, we could estimate the proportion from the total volume of the prepared dish consumed by the respondent and thus also the proportion of each individual ingredient. In addition, we compiled a database with average recipes for the local staple preparations *fufu* (cassava and/or maize flour and water), *lituma* (cooked and mashed plantains and/or cassava) and *chikwangue* (steamed cassava paste) and the most common composite dishes like beans, amaranth, spinach, sweet potato leaves and cassava leaves. Per dish, 5 local recipes were collected and the average recipe calculated [32]. The average recipes for composite dishes were only used in case no individual recipe could be recorded (out of home consumption, in 8.5% of the recalls).

### Species identification

Species have been collected and identified at the herbarium of the National Botanical Garden of Belgium (BR) during previous ethnobotanical research. For the description of species' uses and herbarium references we refer to Termote et al. [25], [26].

### Food composition table

Apart from a very old preliminary table [35], no recent food composition table exists for DRC. To convert ingredients into their nutrient levels, the food composition table for Tanzania [36] was used as basis and completed for lacking food items using the USDA nutrient database (www.nal.usda.gov/fnic/foodcomp/search). For a number of rare or wild foods, other references were used (Kengue [37] for safou (*Dacryodes edulis*); Cunningham & Wehmeyer [38] for palm wine; Eyo et al. [21] and Isong et al. [22] for ‘fumbwa’ *Gnetum africanum*; Enujiugha & Ayodele-Oni [39] for *Tetracarpidium conophorum* and Leung [40] for other wild species). Values for nutrients which were still lacking were replaced with data on similar foods in the food composition table. Where needed, nutrient contents of raw foods were corrected for cooking processes according to the USDA guidelines [41].

### Data analysis

Food intake data from the 24 h recall were entered and processed in the Lucille food analysis software (Ghent University, Gent, Belgium, www.foodintake.ugent.be) and usual food group and nutrient intake distributions were generated using the multiple source method [42], [43]. This method allows eliminating the intra-person variation of the nutrient intake. The distributions generated were adjusted for ‘interviewer’ and ‘recall day’. We omitted pregnant or lactating women in the analyses of nutrient intakes, because their energy and nutrient needs are higher to compensate for pregnancy or lactation.

Statistical analyses were performed in SPlus 8.1 (TIBCO software Inc., Palo Alto, California, USA). χ^2^-tests were used to compare proportions of women consuming different foods and food groups between the 3 samples and ANOVA with Tukey post hoc-tests to compare energy contributions of food groups and nutrient intakes between the 3 samples. Comparisons of micro-nutrient intake were adjusted for total energy intake. The percentage of non-pregnant non-lactating women with micro-nutrient intakes below the RDA for adult women (FAO & WHO 2004) were calculated for the 3 samples. The lowest bio-availability for iron (5%) and zinc (15%) was used as the diets recorded were predominantly plant-based [32]. To assess the nutritional contribution of WEP in the diet, the sample was split in WEP-consumers (eating more than 10 g of WEP, the semi-wild *safou* included, in at least one of both recalls) and non-consumers. Energy contributions of food groups and usual nutrient intakes of both groups were compared in a regression model adjusted for sample design. The significance level was set at 5% for all statistical tests and all tests were two-sided.

## Results

### Participant characteristics

**Table 1** presents the participant characteristics for the three samples. The village sample contained younger women, more lactating women, more women of a polygamous household and with a higher knowledge of WEP. Compared to the other women, they were also more involved in activities such as agriculture, hunting, fishing and collecting WEP, insects or mushrooms.

**Table 1:** Sample characteristics of the food consumption survey.

Characteristics	Kisangani city	Turumbu city	Yaoseko	*P* 2
	*n (%)*	*n (%)*	*n (%)*	
Total number of subjects	241	122	129	
Age (years)^1^	35.0±11.7 ^a^	44.1±14.8 ^b^	30.5±11.0 ^c^	<0.001
Age categories				
<20 years	27(11.2)	9 (7.4)	27 (20.9)	
21–35 years	117(48.5)	28 (23.0)	64 (49.6)	
>35 years	99(41.1)	84 (68.9)	35 (27.1)	
(NA)	(0)	(1)	(3)	
Pregnant	18 (7.5)	5 (4.1)	13 (10.1)	0.19
Lactating	41 (17.0)	9 (7.4)	36 (27.9)	<0.001
Years of schooling^1^	8.2±2.8 ^a^	5.4±3.9 ^b^	4.8±2.3 ^b^	0
Number of WEP known^1^	7.2±2.6 ^a^	8.0±2.3 ^a^	16.2±5.1 ^b^	0
Ethnicity				
Turumbu	6 (2.5)	122 (100)	105 (81.4)	
Tshopo District	137 (56.8)	0	21 (16.3)	
Other	98 (40.7)	0	3 (2.3)	
Marital status				0
Single	16 (6.6)	12 (9.8)	2 (1.6)	
Married (1ste wife)	188 (78.0)	64 (52.5)	92 (71.3)	
Married (2^nd^ or 3^rd^ wife)	15 (6.2)	8 (6.6)	27 (20.9)	
Divorced or widow	22 (9.1)	38 (31.1)	8 (6.2)	
Household members^1^	9.0±4.4	9.4±5.0	8.1±4.4	0.08
Field	56 (23.2)	36 (29.5)	122 (94.6)	0
Garden	195 (80.9)	107 (87.7)	52 (40.3)	0
Cattle raising	105 (43.6)	49 (40.2)	67 (51.9)	0.15
Hunting	6 (2.5)	11 (9.0)	89 (69.0)	0
Fishing	16 (6.6)	15 (12.3)	99 (76.7)	0
Collecting WEP	48 (19.9)	55 (45.1)	120 (93.0)	0
Collecting insects	21 (8.7)	42 (34.4)	120 (93.0)	0
Collecting mushrooms	38 (15.8)	60 (49.2)	123 (95.3)	0

1 Mean with standard deviation;

2 calculated using χ^2^-tests for factor variables; ANOVA for comparison of means, if p<0.05, a Tukey *post-hoc* test was performed, different letters indicate statistically different means at 0.05 level.

### Consumption frequency of food groups and wild foods

Cassava was the most frequently consumed staple food in the three samples (**Table 2**) (consumed in resp. 76.2, 84.3 and 98.8% of the recalls in the overall city, Turumbu city and Turumbu village samples). The second most important staple in the village were plantain bananas. In the city more cereals were consumed, especially rice followed by maize and bread. Cassava leaves constituted the main side dish (more than 50% of all recalls) for women in all samples. Caterpillars form an important part of the diet in all samples as they are abundant during the July–October season (resp. 19.5, 31.5 and 23.1% of the recalls for the general city, Turumbu city and Turumbu village sample). Only 15 WEP (1 wild yam, 2 wild nuts, 4 wild leafy vegetable, 3 wild fruits and 5 wild spices, see table 2 for species identifications) were found in a small number of recalls in the three samples. More consumed was the semi-wild fruit safou (*Dacryodes edulis*) in 30.1% of the recalls in the village and in 19 recalls in the city.

**Table 2:** Proportion of women that consumed food groups and food items^1^.

Food groups and food items	Kisangani (n = 241)	Turumbu city (n = 122)	Turumbu village (n = 129)	*P* 2	
	*n (%)*	*n (%)*	*n (%)*	Kisangani - Tcity	Tcity – Tvillage
**Cereals**	**212.5 (88.1)**	**84 (68.8)**	**32 (24.7)**	**<0.001**	**<0.001**
Rice	151 (62.6)	56 (45.9)	6.5 (5.1)		
Maize	103 (42.7)	33 (27.2)	26.5 (20.7)		
Bread	53 (22.0)	16.5 (13.7)	0.5 (0.4)		
**Roots & tubers**	**183.5 (76.2)**	**103 (84.3)**	**127.5 (98.8)**	**0.09**	**<0.001**
Cassava	173 (71.8)	97.5 (79.9)	127.5 (98.8)		
Plantain	57 (23.6)	48.5 (39.8)	68 (52.7)		
*Wild yam (*Dioscorea* spp.)	0	0.5 (0.4)	0.5 (0.4)		
**Nuts & pulses**	**125.5 (52.1)**	**53.5 (43.9)**	**12.5 (9.7)**	**0.21**	**<0.001**
Peanuts	62.5 (25.9)	38 (31.1)	10 (7.8)		
Cowpea	7.5 (3.1)	2 (1.7)	1.5 (1.2)		
Haricot	47.5 (19.6)	15 (12.4)	0		
Soya	17.5 (7.3)	2.5 (2.1)	0		
Pumpkin seeds	15.5 (6.5)	0	0		
* Wild nuts (*Tetracarpdium conophorum* (Müll.Arg.) Hutch. et Dalziel and *Panda oleosa* Pierre)	0	0	1.5 (1.2)		
**Vegetables**	**233.5 (96.9)**	**112.5 (92.3)**	**115.5 (89.5)**	**0.0075**	**0.45**
Cassava leaves	131.5 (54.6)	66.5 (54.5)	80.5 (62.5)		
Sweet potato leaves	41 (17.0)	24 (19.8)	20.5 (16.0)		
Amaranth	18.5 (7.7)	12.5 (20.1)	9 (7.0)		
Spinach	11.6 (4.8)	4 (3.5)	8.5 (6.6)		
Eggplant	52.5 (21.7)	19 (15.4)	10 (7.8)		
Welsh onion	165 (68.4)	73 (59.9)	44 (34.0)		
Onion	113.5 (47.0)	46 (37.7)	15.5 (12.1)		
Tomato	135 (56.1)	58.5 (48.0)	50.5 (39.1)		
Tomatopaste	87 (36.0)	26.5 (21.7)	2.5 (2.0)		
Celery	98 (40.6)	28 (23.0)	10.5 (8.2)		
* Fumbwa (*Gnetum africanum* Welw.)	0.5 (0.2)	0	0		
* Meye (*Megaphrynium macrostachyum* (Benth.) Milne-Redh.)	0.5 (0.2)	0.5 (0.4)	0		
* Gbedegbede (*Amaranthus dubius* Mart. ex Thell)	0	0.5 (0.4)	0		
* Sese (*Talinum triangulare* (Jacq.) Willd.)	0	0.5 (0.4)	6.5 (5.1)		0.0061
**Fruit**	**55 (23.8)**	**23 (18.8)**	**47 (36.3)**	**0.27**	**<0.001**
Avocado	20.5 (8.6)	4.5 (3.9)	1 (0.8)		
Banana	19 (8.0)	10 (8.0)	1 (0.8)		
Papaya	1.5 (0.6)	0	3.5 (2.8)		
Safou (*Dacryodes edulis* (G.Don.) H.J.Lam.)^3^	9.5 (4.0)	8 (6.4)	39 (30.1)	0.26	<0.001
* Tondolo (*Aframomum laurentii* (De Wild. et T.Durand) K.Schum.)	0.5 (0.2)	0	0		
* Sakanu (*Cola bruneelii* De Wild.)	0	0	0.5 (0.4)		
* Bombi (*Anonidium mannii* (Oliv.) Engl. et Diels)	0	0	4 (3.1)		
**MPO**	**49.5 (20.5)**	**20.5 (16.8)**	**46 (35.5)**	**0.14**	**<0.001**
Bush meet fresh	1.5 (0.6)	1 (0.8)	6 (4.7)		
Smoked bush meet	26.5 (11.1)	13 (10.7)	36.5 (28.1)		
**Fish**	**149.5 (62.0)**	**55.5 (45.3)**	**55 (42.6)**	**<0.001**	**0.88**
Fresh fish	16.5 (6.9)	9 (7.3)	22.5 (17.6)		
Salted fish	74 (30.8)	30.5 (24.8)	22 (17.2)		
Smoked fish	75 (30.8)	24.5 (20.1)	19.5 (15.3)		
**Egg**	**3.5 (1.5)**	**2.5 (2.1)**	**1 (0.8)**		
**Milk&milkproducts**	**38 (15.7)**	**12 (9.8)**	**2 (1.6)**	**0.051**	**<0.001**
Milk	37.5 (15.5)	12 (9.8)	2 (1.6)		
**Oil&fat**	**236.5 (98.1)**	**116.5 (95.6)**	**125.5 (97.3)**	**0.0486**	**0.31**
Palm oil	223.5 (92.7)	114.5 (94.0)	125.5 (97.3)		
Vegetal oil	41.5 (17.2)	15.5 (12.6)	1.5 (1.2)		
**Mushrooms**	**7.5 (3.1)**	**3.5 (2.9)**	**12.5 (9.8)**	**0.58**	**0.0028**
**Caterpillars**	**47 (19.5)**	**38.5 (31.5)**	**30 (23.1)**	**0.0093**	**0.22**
**Sugars**	**214.5 (51.6)**	**51 (41.8)**	**49.5 (38.3)**	**0.11**	**0.50**
Sugar	117.5 (48.7)	49.5 (40.4)	46 (36.6)		
Soft drinks	11.5 (4.8)	2 (1.7)	0		
**Miscellaneous**	**238.5 (99.0)**	**119.5 (97.8)**	**128.5 (99.6)**		
Wild spices	2.5 (1.0)	1 (0.8)	11 (8.6)		<0.001
*Longowu (*Hua gaboni* Pierre ex De Wild.)	0	0	7 (5.5)		
*Bofili (*Scorodophloeus zenkeri* Harms)	0	0	1.5 (1.2)		
*Kalafulu (*Cinnamomum zeylanicum* Blume)	1.5 (0.6)	1 (0.8)	1 (0.8)		
*Ketchu (*Piper guineense* Schumach. et Thonn.)	1 (0.4)	0	1.5 (1.2)		
*Kelele (*Hymenocardia ulmoides* Oliv.)	0	0	0.5 (0.4)		

1 Only food items consumed by at least 5% of a group are reported, except for WEP. All WEP consumed in this study are shown; they are preceded by an asterisk. For WEP herbarium references we refer to Termote et al. (2010, 2011).

2 The Turumbu living in the city were compared with the overall city sample and the Turumbu from the village were compared with the Turumbu from the city. χ^2^-tests were performed for all food groups and wild food items which were consumed by at least 10 persons over the two samples compared.

3 Safou (*Dacryodes edulis*) occurs native in Central Africa. This species is cultivated on a small scale around the homesteads, but also harvested from the wild. It can be considered as semi-wild.

### Energy contribution of food groups and wild foods

**Table 3** shows the energy contributions of 14 food groups and wild foods for the 3 samples. WEP contributed marginally to the energy intake in the 3 samples, except for safou in the village sample (4.8%). In the village, safou contributed more to total energy intake than the meat/poultry/offal, fish, nuts and pulses, vegetables, sugars or caterpillars food groups. The village diets are mainly characterized by a high consumption of roots and tubers (especially cassava, 45.4%), vegetables and a high energy contribution of oils and fat (36% principally from palm oil). City diets are more composed of a mix of roots/tubers (17.5%), cereals (25%) and vegetables and have also a high energy contribution from oils and fats (33%). Sugar energy contribution increases significantly from the Turumbu village sample (1.6%) over the Turumbu city sample (3.1%) to the overall city sample (4.7%). The energy contribution of caterpillars is rather low in comparison with the proportion of women who consume this food group (Table 2) and not significantly different over the three samples.

**Table 3:** Energy contribution of food groups and wild foods per sample^1^.

Food group	Kisangani city		Turumbu city		Turumbu village		*P* 2
	Energy (*kcal*)^3^	% total energy^4^	Energy (*kcal*)^3^	% total energy^4^	Energy (*kcal*)^3^	% total energy^4^	
Cereals	539.9±210.9 ^a^	25.0	355.2±177.5^b^	19.7	39.3±81.6 ^c^	2.1	0
Roots and tubers	383.4±192.6 ^a^	17.5	401.6±168.5 ^a^	22.3	847.7±345 ^b^	45.4	0
- Wild yam	-5		-		-		
Nuts & pulses	170.5±111.8 ^a^	7.8	139.5±164.7 ^a^	7.7	19.1±70.9 ^b^	1.0	0
- Wild nuts	-		-		-		
Vegetables	61.2±23.5 ^a,b^	2.8	57.6±25.4 ^a^	3.1	62±24.8 ^b^	3.3	0.055
- Wild vegetables	-		-		2.2±7.5	0.1	
Fruits	39.8±61.9 ^a^	1.8	30±49.5 ^a^	1.7	95.8±94.1 ^b^	5.1	0.001
- Wild fruits	-		-		9±40.7	0.5	
- Safou	12.1±51 ^a^	0.6	11.7±36.7 ^a^	0.6	89.6±107.2 ^b^	4.8	0
Meat/Poultry/Offal	58.5±93.6 ^a^	2.7	32±82.7 ^b^	1.8	27.9±33.7 ^b^	1.5	0.0004
- Bush meet fresh	-		-		5.7±27.6	0.3	
- Smoked bush meet	17.7±50.3	0.8	9.9.±26.2	0.6	19.9±26	1.0	0.11
Fish and fish products	41.8±35 ^a^	1.9	30.7±34.9 ^b^	1.7	21±23.2 ^c^	1.1	<0.001
Eggs	-		-		-		
Milk/milk products	16.4±40.1 ^a^	0.8	11.6±38.7 ^a^	0.6	0.5±3.8 ^b^		0.0001
Oils and Fats	719.6±196.1 ^a^	33.0	623.8±261.2 ^b^	34.6	663.4±236.4 ^a,b^	35.5	0.0004
Sugars	101.9±89.8 ^a^	4.7	56.4±72.7 ^b^	3.1	29.4±35.3 ^c^	1.6	0
Miscellaneous	18.7±30.6	0.9	16.9±53.2	0.9	31.7±84.4	1.7	0.054
- Wild spices	0.2±1.7	<0.1%	-		0.4±2.4	<0.1%	
Mushrooms	0.4±1.9 ^a^	<0.1%	0.6±2.8 ^a,b^	<0.1%	1.4±3.7 ^b^	0.1	0.0034
Caterpillars	13.5±27.5	0.6	16.2±19.1	0.9	14.9±23.6	0.8	0.59

1 All values are usual intake means ± standard deviation, with adjustment for *recall day* and *interviewer*;

2 ANOVA comparison of means. If p<0.05, a Tukey *post-hoc* test was performed, different letters indicate statistically different means at 0.05 level;

3 1 calorie = 4.1868 Joule.

4 expressed as percentage of total energy intake;

5 “-” indicates that the energy contribution from these foods was insignificant.

Subsequently, we compared the energy contribution of the different food groups between women who consumed WEP and those who did not. The overall city sample counted 20 WEP consumers (safou included), the Turumbu city and Turumbu village sample resp. 18 and 72. After correcting for sampling, WEP consumers consumed significantly more fruits and roots & tubers than non WEP consumers (**Table 4**).

**Table 4:** Energy contribution of food groups for WEP consumers and non WEP consumers^1^.

Food group	WEP consumer^2^ (n = 110)		Non WEP consumer (n = 382)		Difference of means^3^	*P* 4
	Energy (*kcal*)^5^	% total energy^6^	Energy (*kcal*)^5^	% total energy^6^		
Cereals	165.8±234.6	8.2	419.6±255.7	20.9	−27.3	0.21
Roots and tubers	753.6±357.3	37.3	439.4±257.4	21.0	106.3	<0.001
Nuts & pulses	65.9±125.1	3.3	139.6±132.9	7.0	−0.77	0.96
Vegetables	63.7±22.3	3.1	60.6±25.0	3.0	0.93	0.76
Fruits	132.9±83.7	6.6	28.8±51.3	1.4	97.4	<0.001
Meat/Poultry/Offal	32.0±65.0	1.6	47.3±84.1	2.4	−3.4	0.42
Fish and fish products	29.4±33.8	1.5	34.8±33.2	1.7	5.0	0.21
Eggs	-7		-			
Milk/milk products	3.2±3.2	0.2	13.3±38.3	0.7	−3.4	0.73
Oils and Fats	662.5±227.5	32.8	686.4±227.6	34.3	−7.8	0.78
Sugars	42.2±52.5	2.1	80.0±85.6	4.0	−8.1	0.38
Miscellaneous	21.3±24.7	1.1	21.8±61.3	1.1	−9.4	0.17
Mushrooms	1.0±3.3	/	0.6±2.6	/	−0.076	0.82
Caterpillars	18.1±24.7	0.9	13.5±24.5	0.7	5.5	0.07

1 All values are usual intake means ± standard deviation, with adjustment for *recall day* and *interviewer*;

2 people who consumed more than 10 g of WEP in at least one of both recalls (safou included);

3 Model based difference of means (WEP consumer – non WEP consumer), adjusted for the fixed effect *sample*;

4 Model-based adjusted for the fixed effect *sample*.

5 1 calorie = 4.1868 Joule;

6 expressed as percentage of total energy intake;

7 “-” indicates that the energy contribution from these foods was insignificant.

### Usual nutrient intake of non-pregnant/non-lactating women

Women in the overall city sample had a significantly higher daily energy intake (2,102 kcal±444.2) than the women in the Turumbu city (1,715 kcal±599.6) and the Turumbu village samples (1,779.4 kcal±564.9) (**Table 5**). Percentage energy from protein was significantly lower in the village sample (7.6%) than for the other 2 samples (9.2 and 9.4%, resp.). Percentage energy obtained from fat was comparable over the 3 samples.

**Table 5:** Usual daily dietary intakes of non pregnant/non lactating women in Kisangani (city), Turumbu women in Kisangani (city) and Turumbu women in Yaoseko (village)^1^.

Nutrient	Kisangani (n = 182)		Turumbu city (n = 108)		Turumbu Yaoseko (n = 80)		*P* 3
		% women under RDA^2^		% women under RDA^2^		% women under RDA^2^	
Weight (*g*)	1039.64±275.14^a^		872.35±271.83 ^b^		1062.88±354.48^a^		<0.001
Energy (*kcal*)	2102±444.19^a^		1715.08±599.57 ^b^		1779.37±564.85^b^		<0.001
Energy density (*kcal/100 g*)	205.47±23.0^a^		196.13±26.21 ^b^		169.34±21.9 ^c^		<0.001
Energy from protein (*%*)	9.24±2.13^a^		9.36±2.1 ^a^		7.56±1.98 ^b^		<0.001
Energy from lipids (*%*)	44.78±5.42		46.19±6.4		44.18±8.06		0.0686
Total carbohydrate (*g*)^4^	260.79±64.1 ^a^		211.71±64.82 ^a^		241.62±94.55 ^b^		<0.001
Fibre (*g*)^4^	22.48±8.73		17.59±8.76		18.81±7.56		0.4021
Vitamin A (*µg RE*)^4^	4240.06±898.37 ^a^	0	3886.47±764.4 ^b^	0	4301.83±768.44 ^b^	0	<0.001
Vitamin C (*mg*)^4^	89.39±23.46 ^a^	3.85	86.17±29.34 ^b^	5.56	165.61±74.22 ^c^	0	<0.001
Thiamine (*mg*)^4^	1.03±0.27 ^a^	63.19	0.95±0.36 ^b^	72.2	1.07±0.41 ^c^	61.25	<0.001
Riboflavin (*mg*)^4^	2.07±0.73 ^a^	3.85	2.55±1.88 ^b^	7.41	2.52±2.02 ^b^	13.75	<0.001
Niacin (*mg*)^4^	9.12±2.87 ^a^	93.4	8.08±3 ^b^	96.3	7.44±2.76 ^a^	97.5	<0.001
Vitamin B-6 (*mg*)^4^	1.73±0.51^a^	24.18	1.55±0.43 ^b^	31.48	2.40±1.1 ^c^	21.25	<0.001
Folate (*µg*)^4^	219.18±58.84 ^a^	100	202.9±65.88 ^b^	100	238.08±86.34 ^c^	93.75	<0.001
Vitamin B-12 (*µg*)^4^	1.44±0.58 ^a^	93.4	1.28±1.49 ^a^	87.03	0.6±0.57 ^b^	97.5	<0.001
Calcium (*mg*)^4^	406.23±104.98 ^a^	100	384.87±138.13 ^b^	99.07	541.91±245.64 ^c^	95	<0.001
Iron (*mg*)^4^	11.89±3.67 ^a,b^	100	8.93±2.89 ^a^	100	10.42±4.22 ^b^	100	0.0154
Zinc (*mg*)^4^	6.46±2.1 ^a^	91.8	5.04±1.8 ^a^	99.07	3.89±1.9 ^b^	97.5	<0.001
Alcohol (*g*)^4^	0.99±5.7		0.31±2.32		1.37±5.48		0.3143

1 All values are usual intake means ± standard deviation, with adjustment for *recall day* and *interviewer*;

2 % of women under RDA, recommended daily allowances for adults [24];

3 ANOVA comparison of means. If p<0.05, a Tukey *post-hoc* test was performed. Different letters indicate statistically different means at 0.05 level;

4 *P* adjusted for total energy intake in the model as described by [55].

The Turumbu women in the city had significantly lower relative intakes for vitamin A, vitamin C, thiamine, niacin, vitamin B-6, folate and calcium than the women in the overall city sample and higher intakes for riboflavin. The Turumbu in the village had significantly higher intakes for vitamin C, thiamine, vitamin B-6, folate, calcium and iron and lower intakes for niacin, vitamin B-12 and zinc than the Turumbu women in the city.

When comparing usual micro-nutrient intakes with the RDA for adult non pregnant non lactating women, more than 75% of the women in all samples had intakes below the RDA for niacin, folate, vitamin B-12, calcium, iron and zinc. However, most women in all samples had vitamin A, vitamin C and riboflavin intakes above the RDA.

Subsequently, we compared women who ate WEP (including safou) with those who did not (**Table 6**). Corrected for energy intake, WEP-consumers had significantly higher intakes of vitamin A, vitamin C, vitamin B-6 and calcium and relatively lower intakes for riboflavin.

**Table 6:** Usual daily dietary intakes of non pregnant/non lactating WEP-consumers and non-consumers (safou included)^1^.

Nutrient	WEP consumers^2^ (n = 78)		Non WEP consumers (n = 310)		Difference of means^3^	*P* 4
		% women under RDA		% women under RDA		
Weight (*g*)	1099.61±323.98		972.27±291.16		125.4	0.0026
Energy (*kcal*)	1975.7±552.48		1915.98±545.52		213.6	0.0037
Energy from protein (*%*)	8.92±2.44		8.93±2.14		1.0	<0.001
Energy from lipids (*%*)	43.25±6.81		45.5±6.15		−2.3	0.0101
Total carbohydrate (*g*)^5^	262.64±85.97		238.28±70.52		35.6	0.18
Fibre (*g*)^5^	23.23±8.26		19.65±8.77		6.1	<0.001
Vitamin A (*µg RE*)^5^	4247.8±802.44	0	4130.87±863.32	0	63.8	<0.001
Vitamin C (*mg*)^5^	150.79±72.01	0	92.49±36.26	3.23	28.7	0.0025
Thiamine (*mg*)^5^	1.1±0.35	56.4	1.0±0.32	68.39	0.091	0.12
Riboflavin (*mg*)^5^	2.2±1.5	10.26	2.32±1.46	5.16	−0.36	<0.001
Niacin (*mg*)^5^	8.76±2.72	93.59	8.42±3.02	95.81	1.3	0.12
Vitamin B-6 (*mg*)^5^	2.37±0.98	8.97	1.68±0.58	27.74	0.45	<0.001
Folate (*µg*)^5^	245.07±74.92	94.87	211.88±64.82	99.68	28.8	0.79
Vitamin B-12 (*µg*)^5^	0.97±0.76	93.59	1.29±1.02	93.87	0.061	0.83
Calcium (*mg*)^5^	569.14±222.91	93.59	392.82±121.64	100	141.3	<0.001
Iron (*mg*)^5^	11.4±3.79	100	10.6±3.8	100	1.5	0.42
Zinc (*mg*)^5^	5.15±2.25	94.87	5.63±2.22	95.81	0.7	0.25
Alcohol (*g*)^5^	0.88±4.62		0.88±5.04		−0.31	

1 All values are usual intake means ± standard deviation, with adjustment for *recall day* and *interviewer*;

2 people who consumed more than 10 g of WEP in at least one of both recalls (safou included);

3 Model based difference of means (WEP consumer – non WEP consumer), adjusted for the fixed effect *sample*;

4 Model-based adjusted for the fixed effect *sample*;

5 Adjusted for total energy intake in the model as described by [55].

## Discussion

The main finding of this study was that WEP were rarely consumed and do not contribute substantially to diets in this high biodiverse region. In total, only 15 wild species figured in a small number of recalls (11 species in the village, 7 in the city). The most noteworthy contribution came from the semi-wild fruit *D. edulis*, reported in 30.1% of the recalls and contributing 4.8% of total energy intake in the village. This is surprising as other studies have mentioned indigenous food systems where biodiversity is widely used and thus supposed to provide many essential nutrients and variety in diets [3], [5], [8]. However, until now, only a few studies were able to calculate the complete nutrient contributions of correctly identified wild species [15]. In the communal areas in South Africa, Dovie et al. [44], found that 91% of households harvested and consumed wild edible herbs, with a mean daily consumption of 0.2 kg per household. According to Ogle et al. [45], more than 50 wild edible plants contributed, respectively, 81% and 63% of the daily intake of vegetables during the flood period and rainy season in the Mekong Delta in Vietnam and made important contributions to the intake of carotene, vitamin C, calcium and iron. On average 3 wild vegetable species were consumed over a 7 day food frequency recall period in the Mekong Delta as well as in the Central Highland population in Vietnam [46]. Based on those few available studies and the region's high biodiversity we expected a lot more WEP to be regularly consumed.

The results of this study combined with the high number of WEP known in the region and described in Termote et al. [25], [26] indicates a huge gap between knowledge and effective use of WEP. Fifty wild vegetables, 67 wild fruits and 18 wild nuts besides condiments, tubers, tea substitutes, etc. have been inventoried in the Turumbu, Mbole and Bali communities in Tshopo District. In Yaoseko, the inhabitants described 77 WEP, but only 11 species figured in their diets, in the period of highest availability [25], [26]. The findings of this research indicate that ethnobotanists should find ways to clearly separate knowledge from effective use of plant species. Documenting WEP knowledge before it disappears, investigating how WEP are actually used and identifying the determinants for use is needed to develop strategies to (re)valorize their traditional uses.

Our findings show that there is margin for improvement of the dietary intake in the region by consumption of WEP. WEP-consumers ate more food, had higher intakes of energy and vitamin A, vitamin C, vitamin B-6 and calcium. In general, the Turumbu samples were characterized by a low energy intake. UNDP/UNOPS [27] estimated energy intakes for the Oriental Province in 1996 at 1,758 kcal/adult inhabitant, which is comparable to our estimates. The very precarious nutrition situation in rural DRC was also confirmed in recent surveys [47], [48]. An estimated 17.3% of the women the Province have a BMI<18.5 kg.m^−2^ [47]. We also found a high contribution of fats (33 to 35.5% of total energy intake), exceeding the WHO/FAO recommendations for macronutrient intakes (15 to 30% of total energy from fats [23]. Many side dishes contain large amounts of red palm oil, which seems to be a cheap source of energy. In addition, the diet fell short of various micronutrients in particular niacin, folate, vitamin B-12, iron, zinc and calcium with more than 75% of women having intakes below the RDA. According to the DHS-RDC study [47] 49.2% of women in the Oriental Province were anemic. Torheim et al. [13] previously documented how inadequate intakes of multiple micronutrients are common in sub-Saharan Africa and how the promotion of a diversified diet is more appropriate than providing single micronutrient complements in resource poor environments [13].

One could have expected that the diet's low energy and micronutrients can be complemented by consumption of the ready available WEP at least in rural settings. However, the intake of WEP in our sample was too low to significantly contribute to the dietary intake. The composition of various WEP documented in the dietary assessment could clearly improve dietary intake in terms of energy and micronutrients. *Gnetum africanum*, e.g. is a good source of protein, containing all essential amino-acids and many minerals (Na, K, Ca, Mg, Fe; [21], [22], [40]. Furthermore, wild leafy vegetables can ameliorate dietary diversity and compensate for the lack of pharmacologically-active substances - bio-active components with beneficial effects on health - which cultivated species may have lost during domestication [49], [50].

A possible barrier to WEP-consumption may be the distance to be walked to collect certain species in primary forest or the workload to collect and prepare the WEP. A constraint for wild nut consumption may be the high work load involved in cracking the nuts. In addition, many women reported that they do not know about the nutritional qualities of wild foods and expressed their eagerness to know more about nutritional values of their indigenous plants. So, we are convinced that there is room to increase the consumption of traditional foods if appropriate information is provided. Creating ‘awareness’ of the multiple benefits of WEP through nutritional education, role plays, WEP fairs or recipe exchanges are some possible community interventions [51], [52].

An important strength of this study is the dietary assessment method. In this study, we performed two recalls on non-consecutive days, which is appropriate to correct for intra-individual variance, calculate the usual nutrient intakes for the period with highest WEP availability and to compare means of the three samples and to identify the proportion at risk of inadequate intakes [32], [53]. Since potential underreporting might occur, we went great lengths to obtain all WEP consumed. As extreme intakes are frequent and part of the dietary habits in the study population, we did not exclude extreme intakes as over- or underreporters. Food is acquired from day to day and few households store food at home. In the village, households have food reserves on the field, with erratic accessibility during rains. Labor and financial revenue is unpredictable for urban dwellers as well. On various days, one may obtain large quantities of food from relatives or participate in several festivities, which leads to extreme high intakes. Extreme intakes however, are likely to be compensated for and balanced at population level. Another constraint was the lack of an appropriate food composition table. As Baingana et al. [54], we recommend that a food composition table is developed for DRC for at least the common foods. Despite the limitations however, we are confident to provide a fair representation of the dietary contribution of WEP on a population level in our sample. Although Yaoseko is certainly representative for the rural area in Tshopo District, integrating other ethnical groups in the region where we already inventoried WEP, as well as other countries with other biodiversity conditions, in similar studies would contribute to the current knowledge on biodiversity in diets.

The international community (e.g. FAO, Bioversity International) has stressed the need for nutritional composition data on local varieties of crops and wild foods to tap into the potential of biodiversity for food and nutrition security [2]. Our findings provide critical insights into a highly biodiverse context and illustrate the need to translate knowledge into dietary habits. Before getting indefinitely lost, indigenous knowledge on wild edible species and their use in the diet merits further research to capture the potential of biodiversity and ameliorate diet adequacy. Generating food composition data for WEP and making this information available to local populations in combination with research on ecology, management and (participatory) domestication of WEP to be integrated in fields and homegardens, should be considered as a sustainable way to increase nutrition security in the area.

However, the consumption of WEP alone will not suffice to tackle the nutritional problems encountered by the local populations in Tshopo District. The whole agricultural system has been neglected for decades and needs urgent reorganization (CFSVA, 2008). Policy makers, NGO's and local communities should grasp such reorganization to invest in agroforestry and agrobiodiversity. Integration of local fruit trees and traditional vegetables, tubers and others into agroforestry systems and home gardens for better food security, sustainability and biodiversity conservation should be promoted. In the context of vast agricultural potential of DRC, food-based approaches, integrating WEP and agrobiodiversity in the dietary guidelines, are needed to address food security.

### Conclusion

Despite the precarious nutrition security, urban as well as rural inhabitants in a highly biodiverse region as the Congo-basin, do not valorize their knowledge on WEP to complement their diets. Overall consumption of WEP in the sample was too low to achieve adequate dietary intake or nutrition security. The findings of this study indicate the vast gap between agricultural potential and dietary intake in the region and identifies potential avenues for sustainable food security challenges in sub-Sahara Africa.
